# Directly Visualized Carpal Tunnel Release via a Proximal Mini-Transverse Incision: Surgical Technique and Clinical Outcomes

**DOI:** 10.3390/jcm14093234

**Published:** 2025-05-07

**Authors:** Ahmed Majid Heydar, Mustafa Kürklü

**Affiliations:** Orthopedic and Traumatology Clinic, Memorial Bahçelievler Hospital, Bahçelievler Merkez, Adnan Kahveci Blv. No: 227, 34180 Istanbul, Turkey

**Keywords:** carpal tunnel syndrome, carpal tunnel release, mini-open, transverse incision

## Abstract

**Background/Objectives**: Several complications and drawbacks have been described for the endoscopic release of carpal tunnel syndrome as well as for other traditional open and mini-open release techniques. The purpose of this study was to introduce our newly developed minimally invasive technique and report our surgical outcomes. **Methods**: Seventy-five eligible patients with carpal tunnel syndrome who underwent directly visualized carpal tunnel release via proximal mini-transverse incisions were enrolled. Turkish versions of the Quick-DASH, BCTSQ, and VAS were used to quantify the functional quality and pain intensity before surgery, at the third month, and at the final follow-up. In addition, the maximum preoperative and postoperative grip strength were evaluated. **Results**: At the three-month follow-up, there was a significant improvement in the mean VAS score and Quick-DASH score from 5.2 ± 1.4 and 27.46 ± 7 to 1.4 ± 1.1 and 10.2 ± 3.8, respectively. Additionally, significant improvements were observed in both BCTSQ components, the SSS and FSS, with mean preoperative scores of 3.6 ± 1.2 and 3.8 ± 0.9 and postoperative scores of 1.75 ± 0.6 and 2 ± 1, respectively. The Quick-DASH, BCTSQ scores, and VAS scores were further improved at the final follow-up. The mean grip strength gradually increased from 24.2 ± 8.9 kg to 28.2 ± 2.6 kg at the final follow-up. No complications occurred during or after the operation. **Conclusions**: Directly visualized carpal tunnel release via a proximal mini-transverse incision is a viable option for patients with moderate to severe CTS, as it has shown high levels of satisfaction, improved grip strength, and minimal complications.

## 1. Introduction

Carpal tunnel syndrome (CTS) is a condition characterized by the compression of the median nerve within the carpal tunnel, resulting in symptoms that include pain, numbness, and nocturnal paresthesia involving the median nerve distribution of the hand. CTS is considered to be the most prevalent compressive peripheral neuropathy [1] and may account for 90% of all entrapment neuropathies [2]. The symptoms may proceed to progressive atrophy and significant weakness [3], resulting in serious work disability with consequent psychological and financial hardship for affected individuals [4].

Although conservative treatment of CTS, such as splinting, medication, and local injections, could be attempted for mild and moderate neuropathies as initial treatment [5], strong evidence suggests the superiority of surgical treatment, irrespective of the approach [6]. All surgical treatments involve transecting the transverse carpal ligament, resulting in intracompartmental pressure release and decompression of the median nerve. Transection could be performed under visualization of the whole ligament or in a blinded manner [7]. Visualization can be achieved via direct, endoscopic, or ultrasound-guided methods [8]. Additionally, all of these techniques can be categorized regarding the skin incision length into traditional (more than 4 cm), limited (2–4 cm), mini (1–2 cm), percutaneous (0.4–0.6 cm), and ultraminimally invasive (0.1 cm) approaches [9]. The surgical approach can also be classified as either proximal (antebrachial) or distal (palmar), and the procedure can be carried out by either pulling (retrograde) or pushing (anterograde) the transecting tool [9]. The majority of the literature supports the use of a proximal approach because evidence suggests that the palm contains more dense innervation [10].

The potential complications of traditional open carpal tunnel release, such as residual pain, persistent grip strength loss, slower recovery, and scar tenderness and hypertrophy [11], have led surgeons to search for alternatives. First, the endoscopic approach seemed appealing for hand surgeons. Although several studies have revealed that both open and endoscopic release of carpal tunnels are equally effective at improving outcomes [12,13], the endoscopic approach has recently become increasingly popular due to its early recovery, minimal morbidity, and higher satisfaction score [12,14]. Nevertheless, this procedure is technically demanding and is associated with a higher rate of incomplete transverse carpal ligament release and tendon and transient nerve injury [1,15]. Additionally, its requirement for more expensive equipment and skilled endoscopists increases the procedure’s costs. Ultrasound-guided percutaneous carpal tunnel release has also recently emerged as a viable alternative to more invasive surgical techniques. Nonetheless, there is a relative scarcity of data concerning its long-term outcomes and clinical efficacy in the treatment of severe carpal tunnel syndrome [8].

The small transverse distal flexor crease incision for transverse carpal tunnel release was first inspired by the use of endoscopic carpal tunnel release portals and then utilized in blind mini-open procedures to gain the advantages of the endoscopic approach and avoid its drawbacks. Several devices and instruments have evolved to perform blind release [16,17], but all of them share the same concerns of incomplete release and inadvertent neurovascular injury due to limited visualization [17,18,19]. In an attempt to avoid these risks and complications, we developed new techniques to release the transverse carpal ligament under direct vision with a mini-open transverse flexor crease incision. The purpose of this study was to introduce our minimally invasive technique and report the clinical outcomes of the patients.

## 2. Materials and Methods

Consecutive adult patients suffering from CTS who were admitted to the orthopedic outpatient clinic for carpal tunnel release and who underwent directly visualized carpal tunnel release via proximal mini-transverse incisions as a treatment for carpal tunnel syndrome between 2018 and 2023 were enrolled. Following study protocol acceptance and approval by the local ethical committee, informed written consent was obtained from all patients. Patients with unilateral or bilateral idiopathic carpal tunnel syndrome, confirmed by electrodiagnostic studies, who did not respond to conservative treatment for at least 3 months, were at the core of the study population. Patients who underwent surgical intervention on the contralateral side on different days (usually at 3-month intervals) were regarded as separate patients. A minimum of one year of postoperative follow-up and access to medical records were necessary for inclusion. In contrast, patients with secondary carpal tunnel syndrome or severe symptoms such as continual numbness with advanced thenar muscle atrophy and very poor nerve conduction studies were excluded. To maintain homogeneity, patients with diabetes, hypothyroidism, peripheral neuropathy, or associated cervical radiculopathy were also excluded. The severity of carpal tunnel syndrome was graded according to nerve conduction study findings using the American Association of Electrodiagnostic Medicine (AAEM) criteria (Table 1) [20]. A history of trauma and atypical demographics raised the suspicion of secondary CTS and mandated other imaginary investigations, such as X-ray, ultrasonography, or MRI [21]. Typically, middle-aged female patients with increased body mass index who have bilateral involvement are typically diagnosed with idiopathic CTS [22].

All patients were re-evaluated within two weeks and after three months of their respective surgeries. Further clinical reviews were scheduled as needed. To ensure complete follow-up, patients were invited to attend a clinical re-evaluation at least one year after their operation. Patient demographic information, preoperative electromyographic findings, surgical techniques, clinical outcomes, and complication rates were reviewed.

### 2.1. Surgical Technique

**Figure 1:**
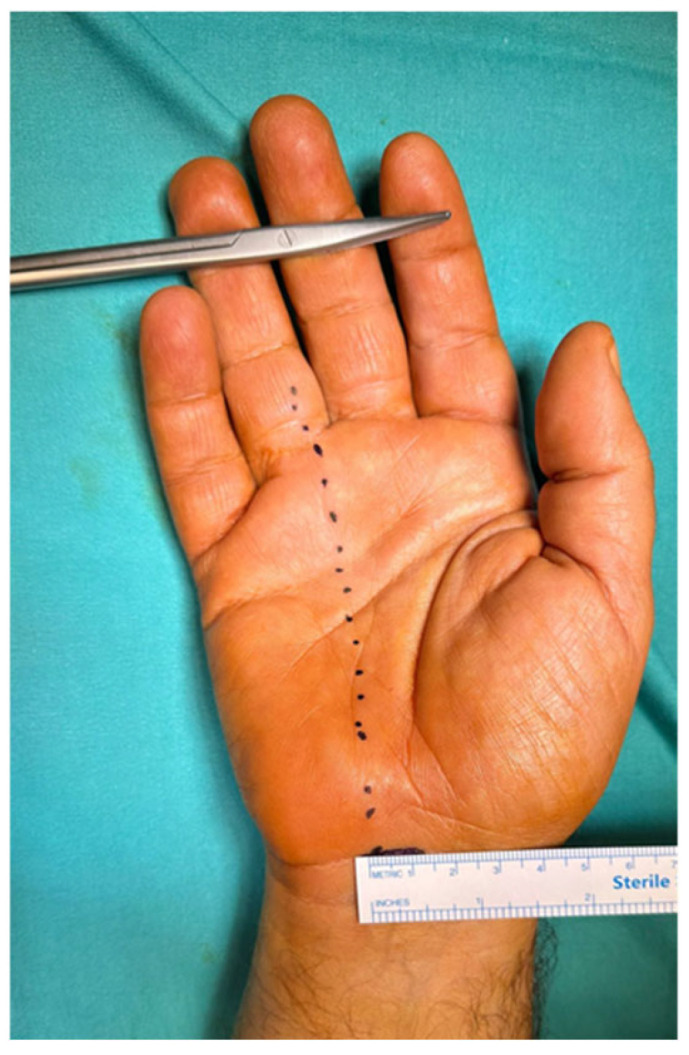
An image of the volar aspect of the wrist showing the localization and size of the proximal mini-transverse incision for carpal tunnel release.

Surgical intervention was performed in the operating room under local anesthesia with conscious sedation (monitored anesthesia care). An upper limb tourniquet was applied, and skin marking was performed. The intersection of a line drawn along the radial border of the ring finger and the distal wrist crease is the ulnar end of the transverse incision (Figure 1), whereas its intersection with the Kaplan cardinal line represents the distal edge of the transverse carpal ligaments. The hand is positioned with a wrist dorsiflexion of 30° over a towel roll.

A 1 cm incision was made, and blunt dissection of the subcutaneous tissue in the direction of the incision was carried out. If a tendon of the palmaris longs was encountered, it was retracted radially. The transition between the antebrachial fascia and transverse carpal ligament was identified and gently divided by a 15-blade knife to expose the underlying median nerve. A curved hemostat was inserted into the carpal tunnel deep to the transverse carpal ligament and advanced in the drawn vertical line of the radial border of the ring finger in such a way that the tip of the hemostat remained in contact with the undersurface of the transverse carpal ligament. The advancement of the hemostat proceeded until the tip of the hemostat was palpated just distal to the distal edge of the ligament, creating a space between the nerve and the transverse carpal ligament. Similarly, another plane was created superficial to the transverse carpal ligament by blunt dissection. A Senn retractor was used to directly visualize the whole transverse carpal ligament, including the distal edge.

**Figure 2:**
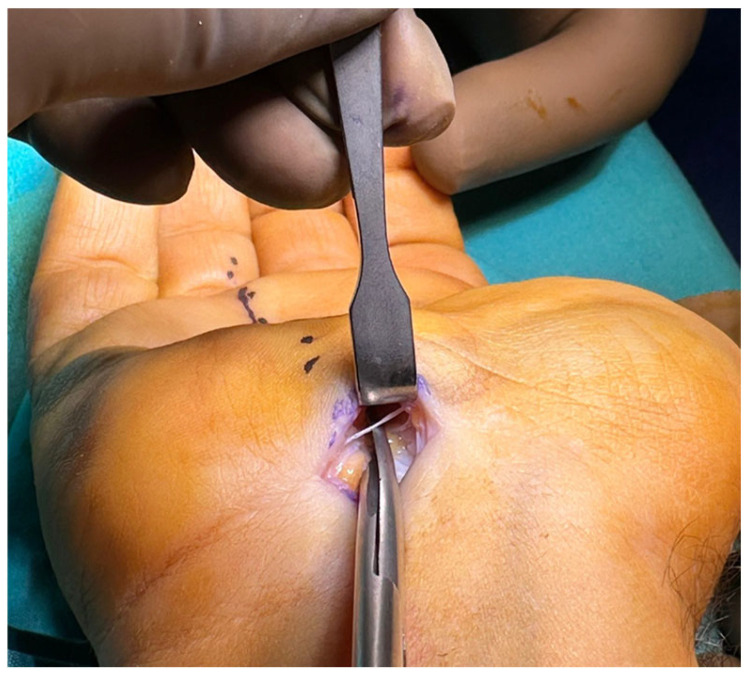
Transverse carpal ligament transection under direct vision by Metzenbaum scissors.

**Figure 3:**
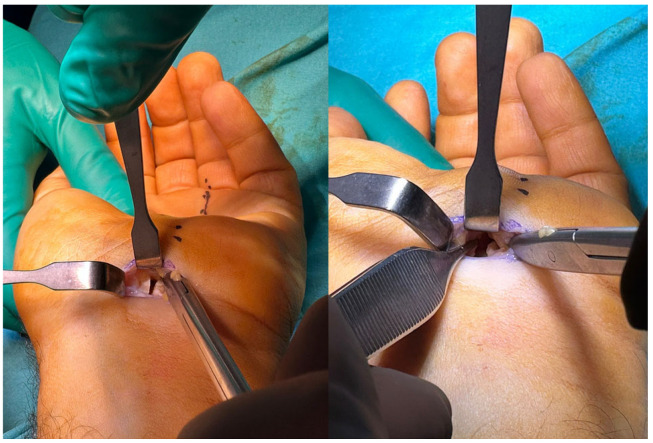
Two different images showing the complete division of the transverse carpal ligament.

Metzenbaum dissecting scissors were used to release the transverse carpal ligament under direct vision. The scissor was directed to the third web space, and the tip of the scissor was pointed ulnarly (Figure 2). The transverse carpal ligament was bisected gradually and cautiously until the tissue resistance suddenly decreased, indicating complete release of the carpal ligament (Figure 3).

**Figure 4:**
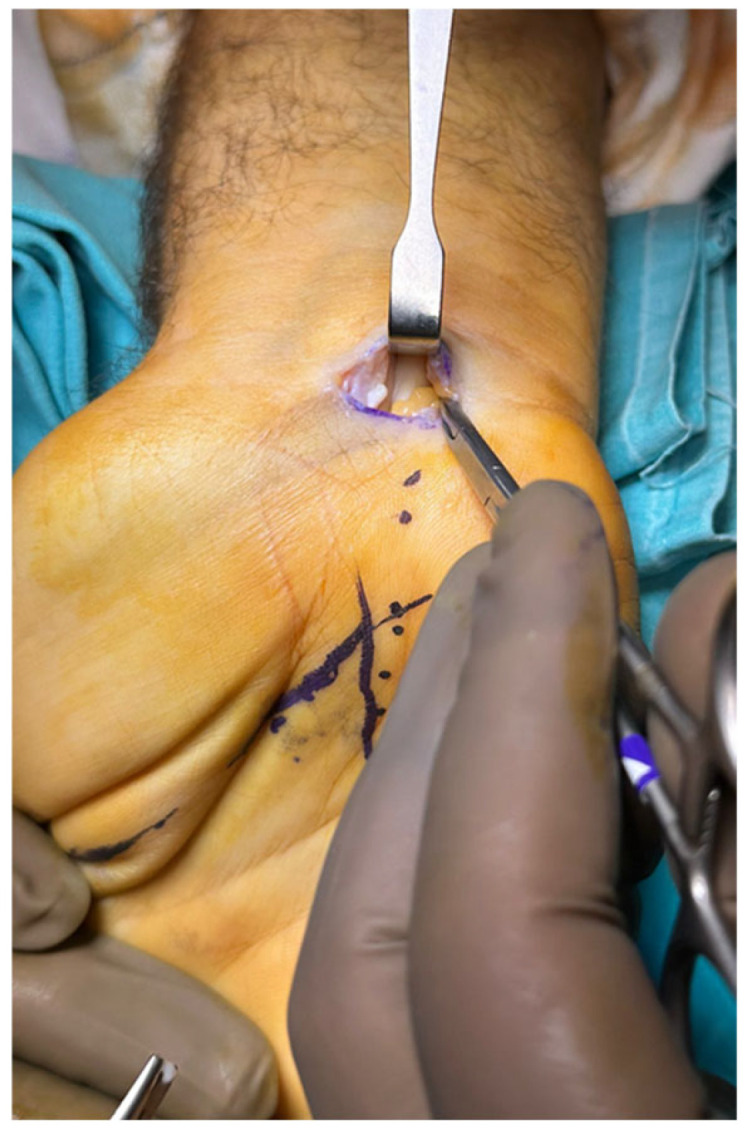
Proximally decompressed median nerve by the retrograde release of the antebrachial fascia.

Maximum care was taken not to direct the scissor radially or dorsally to avoid injuring the recurrent motor branch of the median nerve or proper median nerve, respectively. The smooth passage of the curved hemostat tip from the distal edge of the transverse carpal ligament to the proximal carpal tunnel incision was also used to confirm complete release. The release procedure was only completed when the release of the antebrachial fascia was also achieved (Figure 4).

**Figure 5:**
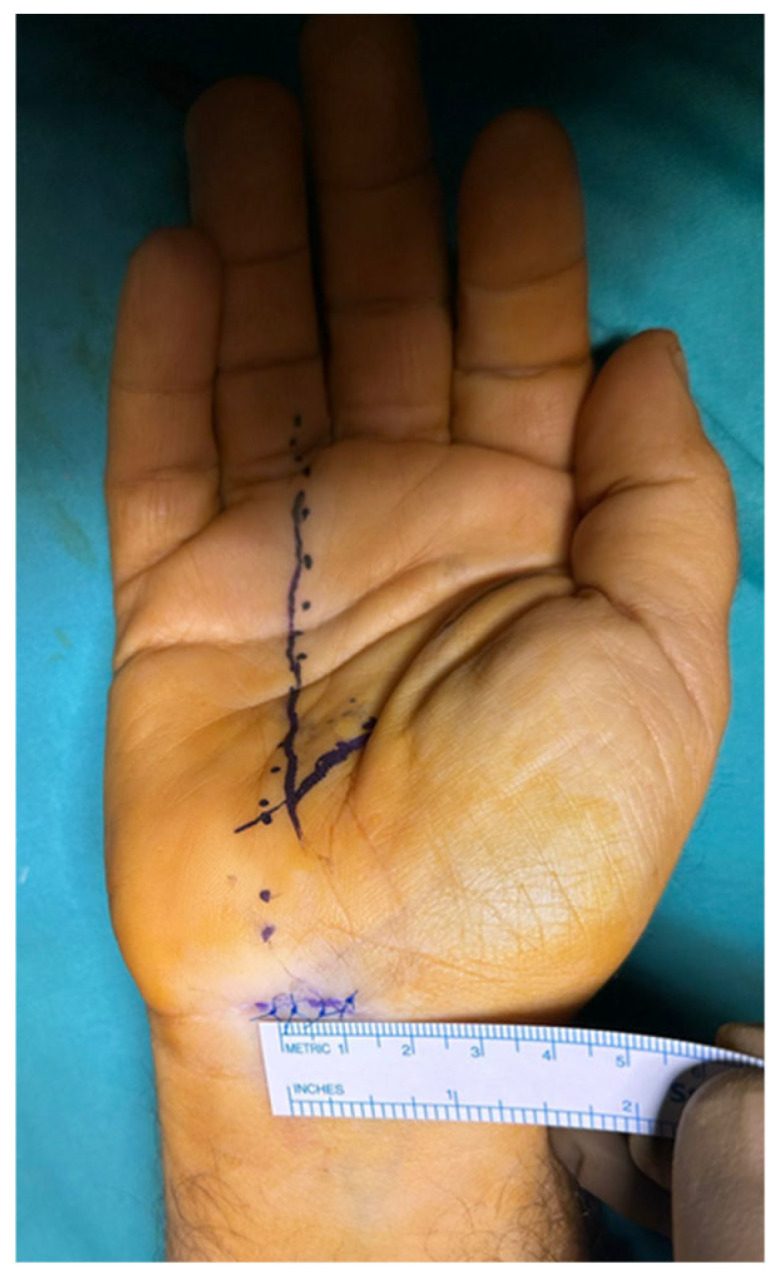
An image showing the incision size (≈1 cm) at the end of the procedure.

A hemostat was used to blunt dissect superficial subcutaneous tissue, and retrograde release of the fascia was also carried out by dissecting scissors under direct vision. Meticulous hemostasis was achieved following tourniquet deflation, the wound was closed with 4 zero nonabsorbable interrupted stitches (Figure 5), and a light compressive dressing was applied.

### 2.2. Postoperative Care

Following pain control, patients were encouraged to actively move their wrist and fingers, and the patients were discharged on the day of the operation. Physical therapy assistance was not needed. Postoperative follow-up occurred at 2 weeks when the stitches were removed, 3 months, and final follow-up, which was at least one year postoperation unless an adverse reaction developed. Clinical evaluations for any tenderness or hypertrophied scar formation at the site of the incision, regression or progression of the signs and symptoms, and outcome measures were recorded.

### 2.3. Outcome Measures

The Turkish version of the Quick Disabilities of the Arm, Shoulder, and Hand Questionnaire (Quick-DASH) [23] and the Boston Carpal Tunnel Syndrome Questionnaire (BCTSQ) were used to quantify functional quality, while the visual analog scale (VAS) (zero means no pain, 10 means maximum pain) was used to assess pain intensity before surgery, at the third month, and at the final follow-up. The BCTSQ has two sections, the Symptom Severity Scale (SSS) and the Functional Status Scale (FSS). These scores were used to compare pain and functional levels between the preoperative and postoperative periods. Additionally, they were used to assess symptom relief or persistence in the third month and symptom recurrence in the final follow-up. The VAS was evaluated using a scale that adheres to rigorous standards for optimal scale size and color selection. A more objective assessment was performed by comparing the maximum preoperative and postoperative grip strength using a Jamar Hydraulic Hand Dynamometer (Model J00105) (Sammons Preston, Bolingbrook, IL, USA). Complications, including intraoperative neurovascular injuries, postoperative wound-related complications, scar tenderness, and pillar pain, were evaluated. Additionally, operated patients were asked about their satisfaction with the surgical outcome and whether they would undergo this operation again at the latest follow-up.

The data were entered into an Excel spreadsheet, and SPSS 1 13.0 (SPSS Inc., Chicago, IL, USA) was used for all analyses. *t*-tests were used to compare the preoperative, postoperative, and final follow-up mean values. *p* < 0.05 indicated statistical significance.

## 3. Results

Surgical carpal tunnel release was indicated for 102 patients, and only 75 patients were eligible for study inclusion. The reasons for exclusion were endocrine disorders (diabetics and hypothyroidism) in 12 patients, loss of follow-up and lack of grip strength measurements in 7 patients, very severe grade (AAEM type 4) in 5 patients, and central neuropathy in 3 patients. Among the included patients, 21 were bilateral, and total surgical release was performed on 96 wrists. They all had either moderate or severe EMG grades (AAEM grade II or III). The basic patient demographics and EMG grades are shown in Table 2.

All of the patients reported improved symptoms immediately after surgery. At the three-month follow-up, there was a significant improvement in the mean VAS score and Quick-DASH score from 5.2 ± 1.4 and 27.46 ± 7 to 1.4 ± 1.1 (*p* < 0.001) and 10.2 ± 3.8 (*p* < 0.001), respectively. Additionally, significant improvements were observed in both BCTSQ components, the SSS and FSS, with mean preoperative scores of 3.6 ± 1.2 and 3.8 ± 0.9 and postoperative scores of 1.75 ± 0.6 (*p* < 0.001) and 2 ± 1 (*p*= 0.001), respectively. While the Quick-DASH and BCTSQ scores improved, the VAS score improved further at the final follow-up (0.7 ± 0.7). The mean grip strength gradually increased from 24.2 ± 8.9 kg to 25.6 ± 6.5 kg in the third month (*p* < 0.001) and 28.2 ± 2.6 kg (*p* < 0.001) at the final follow-up (Table 3).

No complications occurred during or after the operation. There were no instances of neurovascular injury or transient neurapraxia symptoms. There were also no issues with wound healing, joint stiffness, pillar pain, scar tenderness, or excessive scar tissue growth during the follow-up period. None of the patients required additional surgery or experienced recurrent symptoms due to incomplete ligament release. All of the patients expressed satisfaction with the procedure and stated that they would undergo surgery again on the opposite side if necessary. Consequently, 24 patients with bilateral carpal tunnel syndrome underwent a second operation on the opposite side three months after the initial procedure.

## 4. Discussion

The current single-center study investigated the safety and surgical outcomes of directly virtualized carpal tunnel release via a proximal mini-transverse incision. Both the subjective and objective evaluation results demonstrated the benefits of this technique in terms of recovery and the absence of complications. The functional improvements, pain and symptom relief, and grip strength enhancement were of both clinical relevance and statistical significance.

Surgical advancements for carpal tunnel release aim to further decrease the already low complication rate, accelerate recovery, and improve cosmesis. Traditional open carpal tunnel decompression, which is the classic procedure for surgical release of the median nerve, offers the superiority of direct visualization of anatomic structures and consequently minimizes the risk of iatrogenic injury [16]. However, this technique may be linked to specific complications, such as painful scars, neuromas, and neurosensory deficits [24]. The longer incision required for this technique causes a longer healing time and may lead to increased scar tenderness, which negatively impacts the grip strength and quality of life [25]. To achieve superior clinical outcomes while minimizing the incision size, new surgical techniques continue to be developed. A variety of mini-open procedures, such as longitudinal wrist incisions (1–2 cm), mini-transverse proximal incisions, palmar incisions, and double-incision techniques, have been introduced [26]. Data from 1987 and 2012 from surveys of the American Society for Surgery of the Hand (ASSH) members demonstrate a trend of shifting toward less invasive surgical approaches and shorter incision lengths for CTS treatment [25].

Recently, a majority of carpal tunnel release procedures have been performed using a limited longitudinal incision that terminates approximately 2–4 cm distal to the wrist crease [27]. A randomized controlled trial demonstrated superior postoperative recovery and cosmetic outcomes with limited-incision approaches compared to traditional open approaches [28]. Despite the advantages of limited-incision techniques, studies continue to report incision-related morbidities [29]. In a study conducted by Da Silva et al. [30], they demonstrated the presence of unmyelinated nerve fibers derived from the palmar cutaneous branch of the median nerve at the interthenar crease palmar to the flexor retinaculum that are amenable to injury and the development of microscopic neuromas during both traditional open and limited-incision approaches for carpal tunnel release, which could be the cause of scar tenderness. Therefore, attempts have been made to improve surgical outcomes by using further smaller incisions. In a study by Nathan and colleagues, carpal tunnel release procedures were compared between incisions greater than 2.5 cm and smaller incisions. The results showed that the recovery period was shorter with smaller incisions [31]. Additionally, Keramettin et al. demonstrated that pinching improvement, grip strength, and cosmetic results were preferable with a mini-open (1–2 cm) incision compared to traditional open carpal tunnel release [32].

Mini-open incision decompression was implemented using either a longitudinal palmar incision over the flexor retinaculum or a transverse incision over the distal flexor crease. Korkmaz et al. conducted a study comparing mini-open longitudinal and transverse incision approaches and reported that transverse incisions resulted in better cosmesis with less scar formation [33]. Furthermore, the transverse incision retains the fascial layer directly superficial to the transverse carpal ligament, which is primarily derived from the thenar and hypothenar muscle fascia, in addition to the dorsal fascia of the palmaris brevis. The preservation of this fascial layer aids in maintaining the postoperative pinch strength and ensures early return to daily activities [27]. Additionally, studies have shown that no pillar pain or scar tenderness was reported in patients who had mini-transverse incisions compared to small longitudinal or traditional open carpal tunnel release [34,35], which could be attributed to noninterruption of the pillar skin and underlying tissue bridge, which is a feature of the longitudinal incision.

Blind mini-open techniques do not provide direct visualization of the carpal tunnel content, which may be considered a significant drawback despite the reported absence or infrequent complications [7,27,36]. This procedure can be carried out using specialized equipment or routine surgical instruments [7,36], such as hook knives or scissors [17,27]. The most serious complications of the blind mini-incision technique are neurovascular injury and incomplete release [37]. Complete median nerve division after blind mini-open decompression with the Biomet Indiana Tome has been documented in one patient [19]. In our technique, we perform carpal tunnel release under direct vision without the need for any specialized tool or equipment. Decompression under direct visualization can provide complete release, avoiding the persistence or reoccurrence of symptoms and decreasing neurovascular injury risks by easily recognizing and addressing any anatomical variations. Therefore, in our series, no neurovascular complications were observed.

It can be posited that proximal incisions at the distal flexor crease of the wrist do not offer the flexibility to directly visualize the whole transverse carpal ligament and vulnerable neurovascular structures and their variations, which regrettably increases the likelihood of injuring them. However, we propose that the entire transverse carpal ligament, including the distal edge, can be visualized by blunt dissection of the ligament, proper utilization of the Senn retractor, and dorsiflexion of the wrist. In these cases, transection of the entire transverse carpal ligament is performed under direct vision so that injury to neurovascular structures can be prevented.

Carpal tunnel decompression using a mini-transverse incision technique is not ideal for conditions such as revision carpal tunnel syndrome, carpal tunnel syndrome secondary to space-occupying lesions, proliferative tenosynovitis, or bony abnormalities caused by previous trauma. Idiopathic carpal tunnel syndrome is the best candidate for this technique, and the abovementioned conditions should be excluded during preoperative evaluation when the technique is implemented. More extensive exposure, typically with traditional open carpal tunnel release, is recommended.

This research has certain limitations, such as a small sample size that only represents the local community and might not apply to the broader population. Additionally, the study did not involve comparisons with other techniques, as it is a preliminary report. A randomized controlled trial can be conducted to compare the current technique with other methods using reliable outcome measures, such as questionnaires and objective measures. In mini-open-incision release surgery, there is a concern about limited exposure, which may result in incomplete release and damage to surrounding structures. To investigate this further, cadaveric studies can be conducted to determine the risk of collateral damage.

## 5. Conclusions

In summary, we present a new method of performing direct visualized carpal tunnel release through a proximal mini-transverse incision. Our results indicate that this technique is a viable option for patients with moderate to severe CTS, as it has shown high levels of satisfaction, improved grip strength, and minimal complications. Therefore, it could be considered a promising alternative to the traditional open carpal tunnel release procedure.

## Figures and Tables

**Table 1:** The American Association of Electrodiagnostic Medicine (AAEM) classification for grading the severity of carpal tunnel syndrome.

Grade	Criteria
Mild Carpal Tunnel Syndrome	Only sensory peak latency and falling sensory amplitude
Moderate Carpal Tunnel Syndrome	Abnormal median sensory interaction with addition of motor distal latency prolongation
Severe Carpal Tunnel Syndrome	Median motor and sensory distal latency prolongation in addition to sensory and motor amplitude decrease
Very Severe Carpal Tunnel Syndrome	No median sonsory or motor response

**Table 2:** Basic patient demographics and preoperative EMG grades.

Variable	Value
Sex	
Female	48
Male	27
Age	52 ± 7.3 (38–62) years
Side	
Bilateral	21
Right	36
Left	18
Hand dominance	
Dominant	49
Non-dominant	26
Follow-up period	28 ± 4.8 (14–52) months
EMG grade (AAEM grading system)	
Grade 1 (mild)	-
Grade 2 (moderate)	39
Grade 3 (severe)	57
Grade 4 (very severe)	-

**Table 3:** Comparison of pain, functional assessment, and grip strength between preoperative, three-month, and final follow-up values.

		Preoperative	Postoperative			
			3 Months	*p*-Value	Final Follow-Up	*p*-Value *
VAS score		5.2 ± 1.4	1.4 ± 1.1	>0.001	0.7 ± 0.7	0.0019
Quick-DASH Score		27.46 ± 7	10.2 ± 3.8	>0.001	7.4 ± 24.6	0.65
BCTSQ	SSS	3.6 ± 1.2	1.75 ± 0.6	>0.001	1.2 ± 0.7	0.14
	FSS	3.8 ± 0.9	2 ± 1	0.001	1.2 ± 0.6	0.13
Grip strength (in kg)		24.2 ± 8.9	25.6 ± 6.5	>0.001	28.2 ± 2.6	>0.001

* *p*-value: statistical difference between postoperative three-month evaluation and final follow-up values.

## Data Availability

The data presented in this study are available on request from the corresponding author due to patient privacy.

## Abbreviations

The following abbreviations are used in this manuscript:

CTS	Carpal tunnel syndrome
EMG	Electromyography
AAEM	American Association of Electrodiagnostic Medicine
Quick-DASH	Quick Disabilities of the Arm, Shoulder, and Hand Questionnaire
BCTSQ	Boston Carpal Tunnel Syndrome Questionnaire
VAS	Visual analog scale
SSS	Symptom Severity Scale
FSS	Functional Status Scale
ASSH	American Society for Surgery of the Hand
